# P-Rex1 Cooperates With TGFβR2 to Drive Lung Fibroblast Migration in Pulmonary Fibrosis

**DOI:** 10.3389/fphar.2021.678733

**Published:** 2021-07-19

**Authors:** Qing Liang, Yanhua Chang, Jing Liu, Yan Yu, Wancheng Qiu, Jiajia Li, Xu Yang, Guangchun Sun

**Affiliations:** ^1^ Department of Pharmacy, Shanghai Fifth People’s Hospital, Fudan University, Shanghai, China; ^2^ Department of Pathology, The Affiliated Wuxi People’s Hospital of Nanjing Medical University, Wuxi, China

**Keywords:** pulmonary fibrosis, P-Rex1, transforming growth factor β1, fibroblast, migration

## Abstract

Pulmonary fibrosis is a kind of interstitial lung disease with progressive pulmonary scar formation, leading to irreversible loss of lung functions. The TGF-β1/Smad signaling pathway plays a key role in fibrogenic processes. It is associated with the increased synthesis of extracellular matrix, enhanced proliferation of fibroblasts, and transformation of alveolar epithelial cells into interstitial cells. We investigated P-Rex1, a PIP_3_-Gβγ–dependent guanine nucleotide exchange factor (GEF) for Rac, for its potential role in TGF-β1–induced pulmonary fibrosis. A high expression level of P-Rex1 was identified in the lung tissue of patients with pulmonary fibrosis than that from healthy donors. Using the P-Rex1 knockdown and overexpression system, we established a novel player of P-Rex1 in mouse lung fibroblast migration. P-Rex1 contributed to fibrogenic processes in lung fibroblasts by targeting the TGF-β type Ⅱ receptor (TGFβR2). The RNA-seq analysis for expression profiling confirmed the modulation of P-Rex1 in cell migration and the involvement of P-Rex1 in TGF-β1 signaling. These results identified P-Rex1 as a signaling molecule involved in TGF-β1–induced pulmonary fibrosis, suggesting that P-Rex1 may be a potential target for pulmonary fibrosis treatment.

## Introduction

Pulmonary fibrosis (PF) is a chronic interstitial lung disease that leads to progressive destruction of lung architecture and function, characterized by inflammatory microenvironment, interstitial fibroblast proliferation, and deposition of extracellular matrix (ECM). It may be a consequence of cigarette smoking, chemotherapy, and farming/livestock exposure or unknown etiology. Exertional dyspnea and dry cough are the symptoms that the patients generally experience (Lederer et al., 2018). It is often misdiagnosed in the early stage due to the nonspecific, vague, and usually mild initial symptoms. The prevalence of pulmonary fibrosis has been increasing every year. A study collecting data from the US Medicare beneficiaries reported that the number of cases was 494.5 per 100,000, people aged >65 years in 2011 (Song et al., 2019). Pirfenidone and nintedanib, proposed by idiopathic pulmonary fibrosis guidelines, exert anti-fibrotic effects and improve the survival rate after diagnosis (Raghu et al., 2015). However, the long-term treatment efficacy has not been identified. At present, the pathogenesis of pulmonary fibrosis remains elusive. It is believed that the development of pulmonary fibrosis initiates at the early stage of alveolitis and transits to interstitial inflammation. Activated inflammatory cells and interstitial cells secrete transforming growth factor β (TGF-β), matrix metalloproteinases (MMPs), and interleukin, participating in the repair and reconstruction of the lung tissue, eventually leading to pulmonary fibrosis (Wolters et al., 2014; Lederer and Martinez, 2018). However, it is still necessary to explore the pathological mechanism of pulmonary fibrosis.

Multiple mediators contribute to disease progression in pulmonary fibrosis including fibroblasts, epithelial cells, macrophages, cytokines, and matrix. Fibroblasts and myofibroblasts are capable of secreting ECM components and cytokines associated with pulmonary fibrosis, playing central roles in the fibrogenic process. As a key fibrogenic factor, TGF-β1 is associated with the increased synthesis of ECM, enhanced proliferation of fibroblasts, and transformation of alveolar epithelial cells (AECs) into interstitial cells (Saito et al., 2018a). TGF-β1–activated signaling pathways contain canonical Smad-dependent signaling and Smad-independent signaling cascades. Accumulating evidence suggests that cross-regulation between the TGF-β signaling pathway and other canonical signaling occurs in some cases during the progression of diseases, particularly fibrosis (Derynck et al., 2003). Rho-GTPase family proteins are known as important signaling intermediates in controlling intracellular processes including cytoskeleton regulation, cell adhesion, gene transcription, and reactive oxygen species (ROS) formation. It is reported that Rho/Rock cross-talks with TGF-β signaling promote lung fibroblast differentiation (Ji et al., 2014). Activation of Rho-GTPase is regulated by guanine nucleotide exchange factors (GEFs) through facilitating exchange of GDP for GTP (Rossman et al., 2005). PtdIns(3,4,5)P3-dependent Rac exchanger 1 (P-Rex1) is a guanine nucleotide exchange factor (GEF) for Rho GTPase subfamily Rac. We have identified P-Rex1 deficiency attenuated bleomycin-induced mice pulmonary fibrosis based on reduced inflammatory response and decreased lung fibroblast activation (Liang et al., 2016). P-Rex1 acts as a convergence node of G protein–coupled receptor signaling and phosphoinositide 3-kinase (PI3K) signaling. It is primarily involved in the regulation of inflammatory response and neurite differentiation, but also plays a role in endothelial permeability, pulmonary fibrosis, and cancer metastasis (Welch, 2015; Baker et al., 2020; Cai et al., 2020; Ye, 2020). In this study, we further investigated the exact role of P-Rex1 in lung fibroblast activation during fibrogenic processes. Using the P-Rex1 knockdown and overexpression system, we established a novel player of P-Rex1 in mouse lung fibroblast migration. Moreover, we demonstrated that P-Rex1 contributed to pulmonary fibrosis in lung fibroblasts through interaction with TGFβR2. The RNA-seq analysis for expression profiling confirmed the involvement of P-Rex1 in TGF-β1 signaling. Collectively, these findings indicate that P-Rex1 plays a pivotal role in fibrogenic processes and raise the possibility of targeting P-Rex1 for pulmonary fibrosis therapy.

## Materials and Methods

### Clinical Data of Patients With Pulmonary Fibrosis From the Dataset

The P-Rex1 expression patterns in pulmonary fibrosis and normal lung tissues were compared from the NCBI Gene Expression Omnibus (GEO) (http://www.ncbi.nlm.nih.gov/geo). The GSE93606 dataset contained 154 patients diagnosed with IPF and 20 matched controls. The GSE19976 dataset collected lung biopsy samples from eight patients with nodular, self-limiting pulmonary sarcoidosis and seven patients with progressive, fibrotic pulmonary sarcoidosis. The GSE11196 dataset compared the expression profiling by array of lung myofibroblasts from patients with IPF (6 donors) to control (6 donors) using both total and polyribosomal RNA. The GSE40151 dataset was regard to mice treated intratracheally with a single dose of bleomycin or saline.

### Immunohistochemistry

Lung tissue samples from five patients with pulmonary fibrosis and five healthy controls were collected at the Affiliated Wuxi People’s Hospital of Nanjing Medical University. Patients with pulmonary fibrosis were three males and two females. Their age was 42, 75, 67, 54, and 76 years. Healthy controls were four males and one female. Their age was 54, 47, 44, 60, and 68 years. All experimental procedures related to human samples were approved by the Research Ethics Committee of Shanghai Fifth People’s Hospital, Fudan University (2017EC019), and conducted in accordance with the Code of Ethics of the World Medical Association (Declaration of Helsinki). The donors were informed completely and they gave their consent. Paraffin sections were subjected to deparaffinization and antigen retrieval steps. The sections were incubated with primary antibody against P-Rex1 (Cell Signaling Technology, Beverly, MA; 75,706, 1:100) overnight at 4°C, followed by a coloration development with the DAB chromogenic kit (Boster, Wuhan, China). The sections were counterstained with hematoxylin as per the manufacturer’s instructions. The level of stained P-Rex1 was semiquantitatively obtained using Image-pro plus Software.

### Cell Preparation and Construction of P-Rex1 Knockdown and Overexpression System

Mouse lung fibroblasts were isolated and cultured as previously described (Liang et al., 2016). Adult mice were euthanized and lungs were removed and split into small pieces, followed by a collagenase (type IV, Sigma) digestion. Tissue fragments were washed and cultured in DMEM containing 10% FBS with penicillin and streptomycin. Confluent fibroblasts were harvested after 7 days. The use of mice was carried out in accordance with the guideline for ethical review of laboratory animal welfare (China National Standard GB/T 35892–2018). For the knockdown system, short hairpin RNAs (shRNA) were designed specifically targeting P-Rex1 and subcloned into a pLKO.1-puromycin vector. For the overexpression system, full-length P-Rex1 coding sequences were cloned into the pCDH-CMV-MCS-EF1-Puro vector. Afterward, lentiviral particles were produced by cotransfection of packaging plasmids and cloned plasmids to 293T cells. Fibroblasts were infected by the lentiviral particles and screened with puromycin for 48 h to generate stably transfected cells. RT-qPCR analysis and Western blot were used to verify the level of P-Rex1.

### Cell Migration Assay

P-Rex1 knockdown and overexpression fibroblasts were serum deprived overnight. A total of 2 × 10^4^ cells/well in serum-free DMEM were seeded in the transwell upper chamber. The lower chamber contained DMEM with TGF-β1 (10 ng/ml, Peprotech, NJ, United States). After 16 h incubation in the cell incubator, fibroblasts were fixed in methanol and stained with 0.1% crystal violet for 20 min. The migrated cells were counted in five random fields under a 200× objective lens and presented as mean ± SEM.

For the *in vitro* wound-healing assay, P-Rex1 knockdown and overexpression fibroblasts were seeded into six-well plates until confluence. Wounds were created using a standard 200 μL pipette tip. Cells were kept in DMEM containing 2% FBS with or without TGF-β1 (10 ng/ml) for a total period of 24 h. Microphotographs were taken at 0, 12, and 24 h. Cell migration was determined by calculating the reduced area vs. total area.

### Immunofluorescence

Fibroblasts seeded on the coverslips in 24-well plates (2 × 10^4^/well) were serum starved for 12 h at 60% cell density followed by incubation with DMEM containing TGF-β1 (10 ng/ml) or buffer control for 30 min or 1 h. Cells were fixed with 4% formaldehyde in PBS for 10 min and permeabilized with 0.1% Triton X-100 in PBS for 5 min. After blocking with 5% BSA for 1 h, cells were incubated with anti-TGFβR1 antibody (Abcam, E Gottlieb, United Kingdom) overnight at 4°C. The cells were then incubated for 1.5 h at room temperature in the dark with Alexa Fluor 488-conjugated secondary antibody and counterstained with DAPI for 3 min. Images were captured using a confocal fluorescence microscope (Leica Microsystems, Germany).

### Co-Immunoprecipitation

GFP-tagged TGFβR2 plasmid was obtained from Addgene (Cambridge, MA, United States). For heterologous co-IP assay, 293 T cells were transfected with different plasmids (AU5-P-Rex1, and TGFβR2-GFP) or the vector as control. 48 h post-transfection by polyethyleneimine (PEI), cells were lysed with RIPA buffer (Cell Signaling Technology, MA, United States) for 30 min. For endogenous co-IP assay, mouse lung fibroblasts were stimulated with TGF-β1 (10 ng/ml) or buffer control for a total of 2 h. All cell lysates were incubated with a mixture of 3 μg of specific tag antibodies and Protein A/G PLUS-Agarose (Santa Cruz, United States) by rotation overnight at 4°C. The pellets were collected and washed five times, and finally subjected to Western blot analysis to display the results.

### Luciferase Activity Assay

Smad-dependent SBE4-Luc luciferase reporter plasmid was obtained from Addgene. A Dual-Luciferase Reporter Assay kit (Promega, WI, United States) was used to analyze luciferase activity, following the manufacturer’s instructions. Briefly, P-Rex1 knockdown and overexpression fibroblasts were treated with TGF-β1 (10 ng/ml) or buffer control for 16 h and lysed in lysis buffer. Background signal and apparent luminescence activity were measured on a microplate reader (Tecan, Switzerland).

### RNA-Seq Analysis

The RNA-seq analysis was carried out on P-Rex1 knockdown and scramble fibroblasts using next-generation sequencing. Briefly, the total RNA was extracted from P-Rex1 knockdown and scramble fibroblasts using the TRIzol Reagent (Invitrogen) and tested for quality. Illumina Hiseq sequencing was performed. Differentially expressed genes with *p*-value less than 0.05 were obtained. Gene Ontology (GO) enrichment was run using TopGO software. Kyoto Encyclopedia of Genes and Genomes (KEGG) enrichment was analyzed at the KEGG website. Disease annotation function analysis aimed at functional annotation and classification of disease types using the DisGeNET database. Heatmap and bubble chart were performed in R Studio for data visualization.

### Western Blot Analysis

Fibroblasts were plated in six-well plates overnight and challenged with TGF-β1 (10 ng/ml) or control buffer for indicated times. Total proteins of cells were extracted in RIPA lysis buffer. The protein concentration was validated with a BCA kit (pierce, CA, United States). The cell lysate was analyzed by immunoblotting with primary antibodies. Anti-AU5 tag antibody was from GeneTex. Antibodies against phospho-TGFβR2, TGFβR2, phospho-TGFβR1, and TGFβR1 were purchased from Abcam. Antibodies against P-Rex1, phospho-Smad2/3, Smad2/3, GFP, GAPDH, and β-actin were from Cell Signaling Technology. Quantification of Western blots was performed using NIH Image J software.

### Real-Time Quantitative PCR Analysis

Total RNA from fibroblasts was extracted using a RNeasy Mini Kit (QIAGEN, Germany) according to the manufacturer’s instruction. The complementary DNA (cDNA) was prepared using PrimeScript™ RT Master Mix (TAKARA, Shiga, Japan) and subsequently subjected to real-time PCR to quantify the transcripts using SYBR Premix Ex Taq (TAKARA, Shiga, Japan).

### Statistical Analysis

Western blot analysis was determined by comparing the treatment values with control values after normalization against loading controls. Data were expressed as means ± SEM from three independent experiments. One-way ANOVA was performed to evaluate the differences between groups. Student’s *t* test was used for other analyses. Statistical significance was defined as *p* < 0.05.

## Results

### Identification of Induced Expression of P-Rex1 in Pulmonary Fibrosis

**FIGURE 1:**
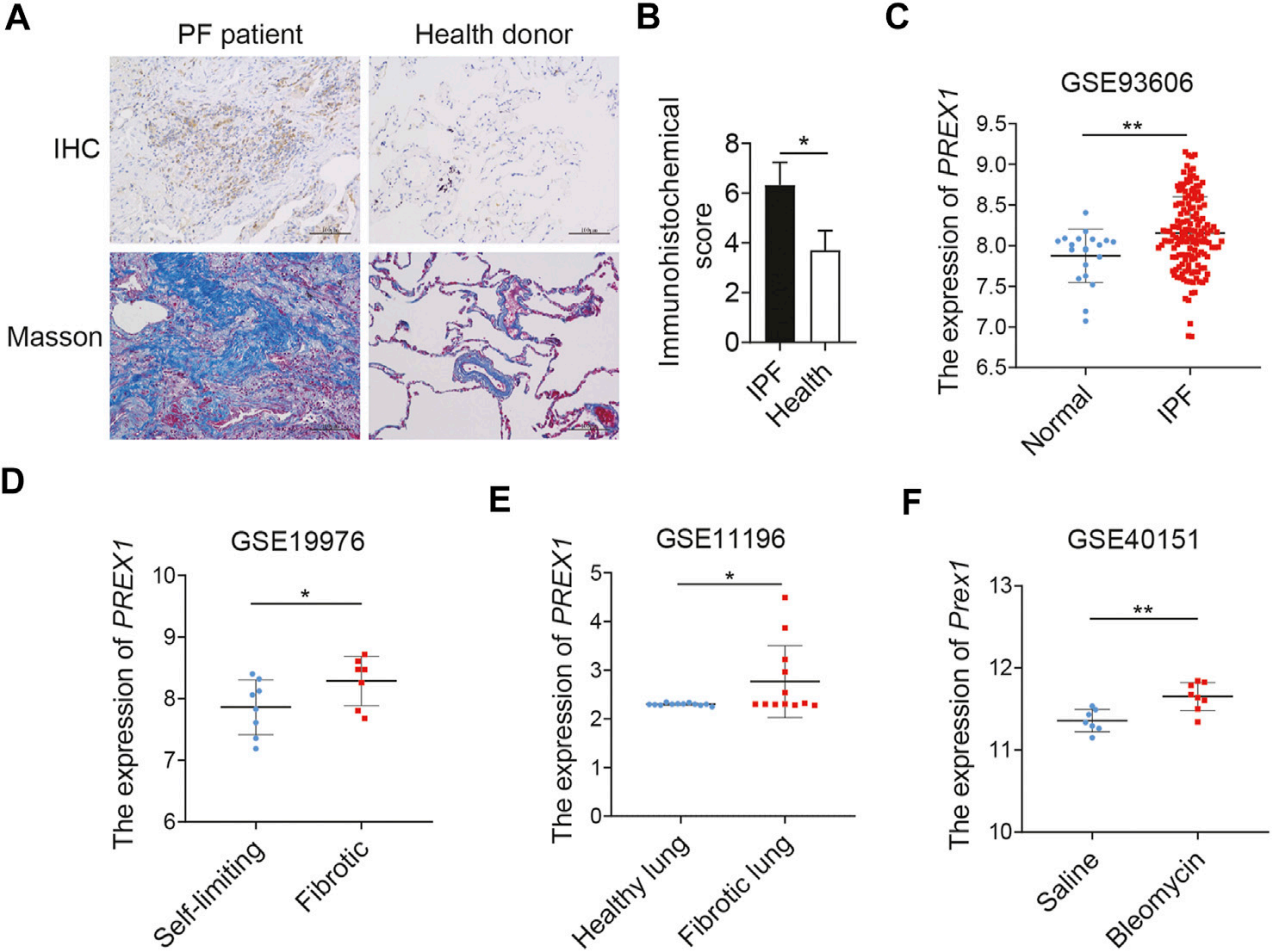
P-Rex1 expression in pulmonary fibrosis. **(A**, **B)** Immunohistochemical staining and Masson’s trichrome staining of lung sections from patients with pulmonary fibrosis and healthy donors. Scale bars = 100 μm, *n* = 5. **(C**–**F)** PREX1 mRNA expression was analyzed between pulmonary fibrosis and non-diseased controls using the public gene expression GEO datasets (GSE93606, GSE19976, GSE11196, and GSE40151) (*p < 0.05, and **p < 0.01).

The level of P-Rex1 expression was first detected in the lung tissues of patients with pulmonary fibrosis. Results obtained from human lung sections collected at the Affiliated Wuxi People’s Hospital of Nanjing Medical University confirmed that the expression of P-Rex1 in fibrosis patients was obviously upregulated compared to that in the normal donors. Histological analysis of lungs from patients with pulmonary fibrosis showed excessive cell proliferation, destruction of alveolar wall, and massive collagen accumulation (Figures 1A,B). Four independent pulmonary fibrosis microarray datasets from public gene expression datasets of the Gene Expression Omnibus database (GEO) were analyzed for PREX1 mRNA levels compared to non-diseased controls (Figures 1C–F). PREX1 was present at a significantly higher transcription level in the peripheral whole blood of patients than normal controls (GSE93606). The lung biopsy sample from patients with nodular, self-limiting disease expressed less PREX1 than patients with progressive, fibrotic disease (GSE19976). Moreover, increased PREX1 mRNA levels were found in lung myofibroblasts from patients with idiopatic pulmonary fibrosis compared to donors using noncontractile matrices (GSE11196). In a bleomycin-induced mice pulmonary fibrosis model, Prex1 transcript level was significantly elevated after bleomycin administration in comparison with saline (GSE40151). These results suggest that P-Rex1 may be involved in the development of pulmonary fibrosis.

### P-Rex1 is Required for Mouse Lung Fibroblast Migration

**FIGURE 2:**
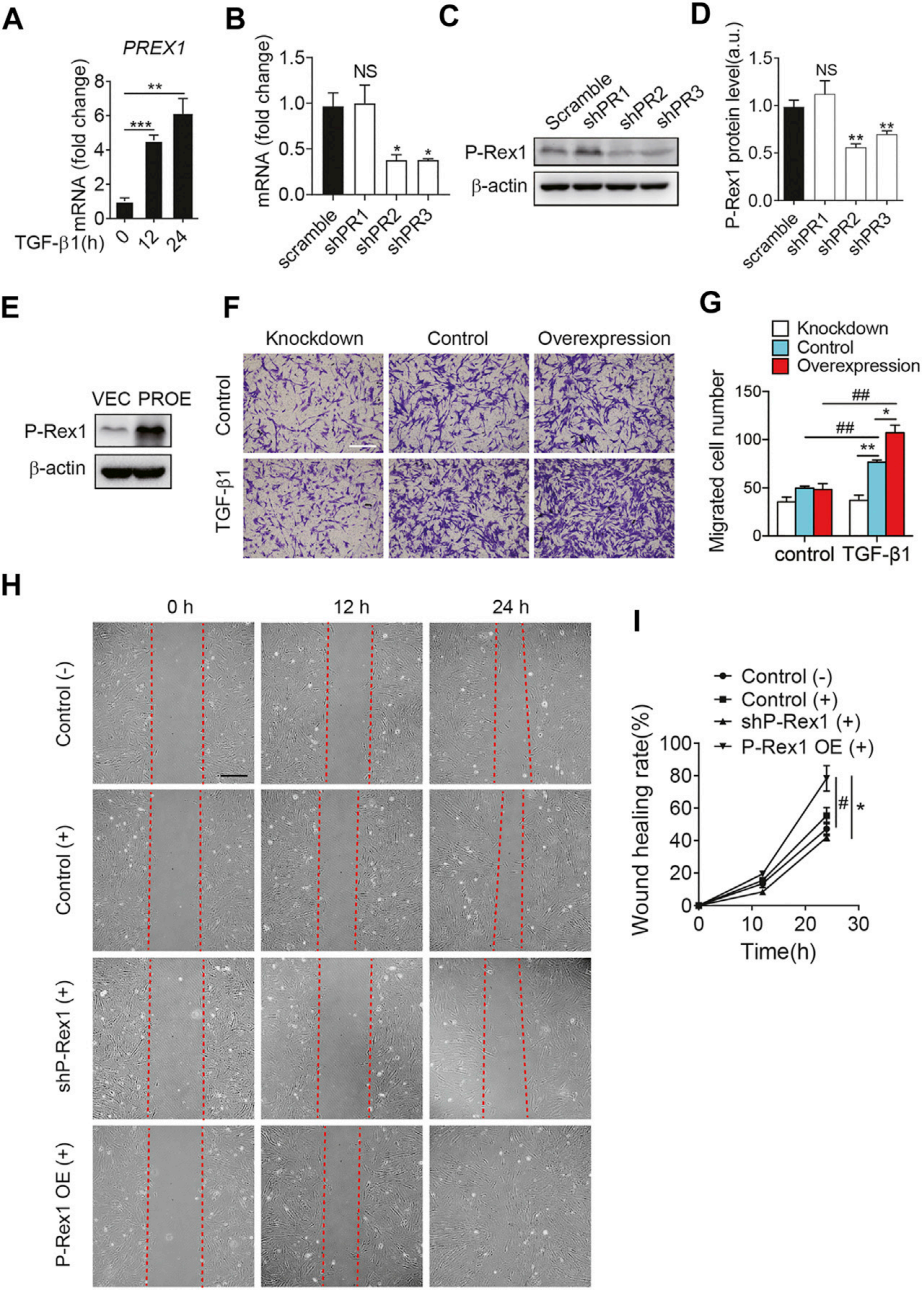
P-Rex1 is involved in mouse lung fibroblast migration. **(A)** The transcript for PREX1 was determined after TGF-β1 stimulation for the indicated time (hour) in mouse lung fibroblasts. Knockdown **(B**–**D)** and overexpression (PROE) **(E)** of P-Rex1 in lung fibroblasts were constructed using recombinant lentiviral transfection. RT-qPCR analysis and Western blot were performed to verify the level of P-Rex1. **(F)** TGF-β1 (10 ng/ml) or buffer control induced cell migration with P-Rex1 knockdown or overexpression fibroblasts. Representative images of fibroblasts migrated through polycarbonate filter membrane are shown, scale bars = 250 μm. **(G)** Analysis of migration in different fibroblasts. **(H**, **I)** Wound closure with P-Rex1 knockdown or overexpression fibroblasts treated with (+) or without (−) TGF-β1 (10 ng/ml) for a total of 24 h. Scale bars = 250 μm. Quantitative data shown are mean ± SEM from three independent experiments (*p < 0.05 and **p < 0.01 comparing P-Rex1 knockdown or overexpression with scrambled control cells; ^#^ *p* < 0.05 and ^##^ *p* < 0.01 comparing TGF-β1–treated cells with untreated cells in the same group).

It is clear that fibroblasts play a critical role in the maintenance of normal tissue repair and pathogenesis of fibrosis responding to a number of stimuli. TGF-**β**1 induces fibroblasts to differentiate into myofibroblasts. Myofibroblasts could synthesize extracellular matrix components and induce TGF-**β**1 autocrine, leading to pulmonary fibrosis (Chanda et al., 2019). At the mRNA level, P-Rex1 was significantly elevated at 12 h after TGF-β1 treatment in mouse lung fibroblasts, suggesting a correlation between P-Rex1 and TGF-β1 signaling (Figure 2A). To further validate the role of P-Rex1 in lung fibroblast activation, stable overexpression and knockdown of P-Rex1 in cultured lung fibroblasts from mice were constructed using recombinant lentiviral transfection. Western blot analysis and RT-qPCR were performed to validate the transcription and expression levels of P-Rex1. One P-Rex1 knockdown clone (Group shPR2) and one P-Rex1 overexpression clone (PROE) as well as the corresponding control cells were obtained (Figures 2B–E). P-Rex1 was usually identified as a GEF for small GTPase Rac, thereby regulating the migration for a certain cell type including neutrophils, neurons, and specific cancer cells. Fibroblast migration was analyzed using a transwell assay and an *in vitro* wound-healing assay. TGF-β1 induction increased the lung fibroblast migration ability. P-Rex1 knockdown fibroblasts exhibited a slower migration rate compared to control cells. Conversely, P-Rex1 overexpression accelerated lung fibroblast migration (Figures 2F,G). A wound-healing assay showed the similar results that P-Rex1 expression increased the wound healing rate; however, knockdown of P-Rex1 visibly slowed down fibroblast migration (Figures 2H,I). Collectively, these data show that P-Rex1 facilitates the motility and migration of lung fibroblasts.

### P-Rex1 Contributes to the Fibrogenic Processes by Acting as a Downstream Mediator of TGF-β1 Signaling

**FIGURE 3:**
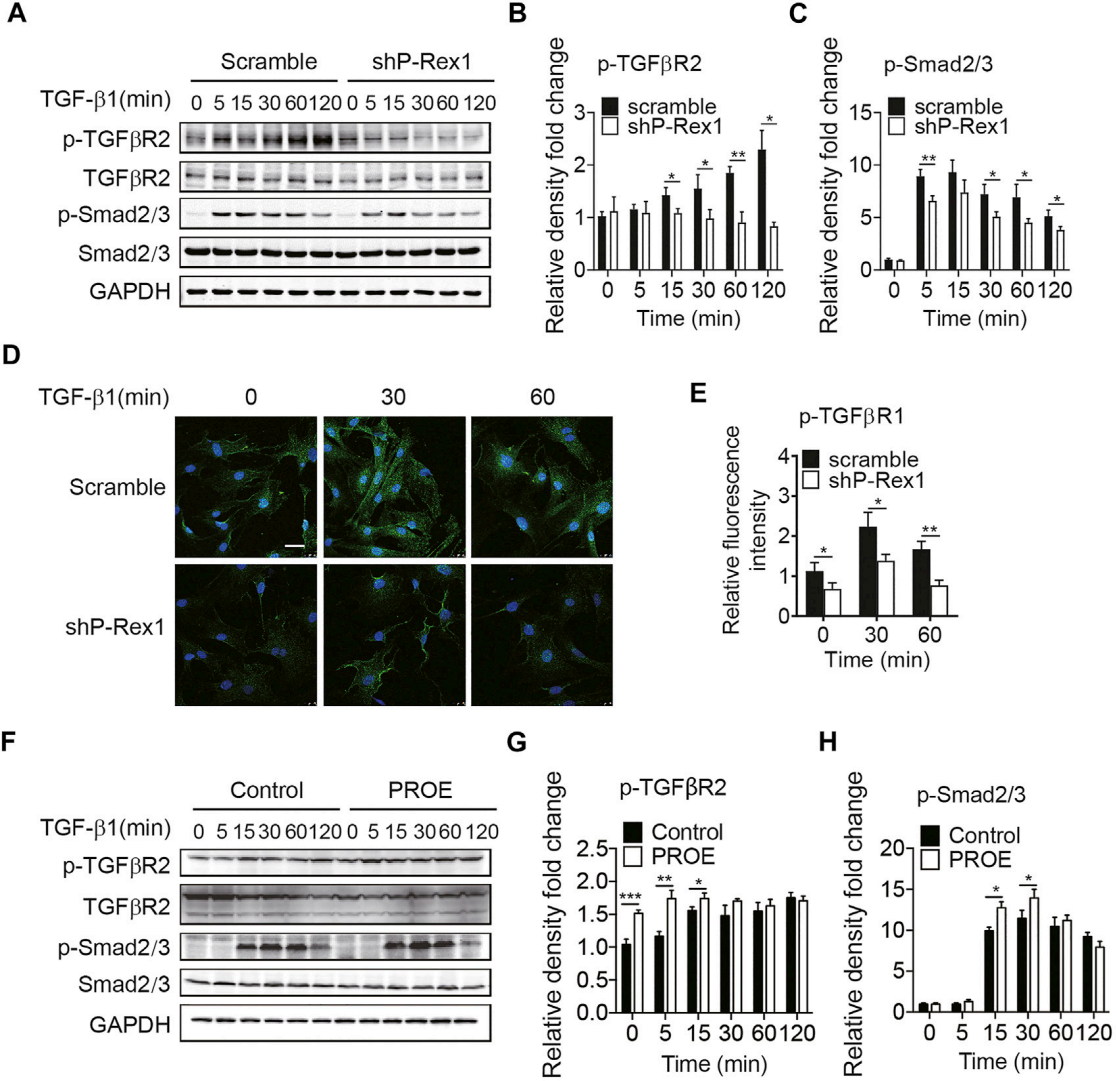
P-Rex1 is downstream mediator of TGF-β1 signaling in fibroblasts. **(A**–**C)** Levels of phosphorylation of TGFβR2 and downstream effector Smad2/3 after TGF-β1 (10 ng/ml) stimulation for the indicated time in P-Rex1 knockdown (shP-Rex1) and scrambled control fibroblasts. **(D**, **E)** Immunofluorescence of TGFβR1 phosphorylation after TGF-β1 (10 ng/ml) treated for the indicated time in different fibroblasts, and signal was quantified. Scale bars = 10 μm. **(F**–**H)** Levels of phosphorylation of TGFβR2 and Smad2/3 after TGF-β1 (10 ng/ml) stimulation for the indicated time in P-Rex1 overexpression (PROE) and control fibroblasts. Quantitative data shown are mean ± SEM from three independent experiments (*p < 0.05, **p < 0.01, and ***p < 0.001).

Given the requirement for P-Rex1 in TGF-β1–mediated fibroblast migration, we sought to determine whether P-Rex1 was involved in TGF-β1 signaling from lung fibroblasts. Downregulation of P-Rex1 in mouse lung fibroblasts abolished the TGF-β1–induced phosphorylation of TGFβR2 and downstream effector Smad2/3 (Figures 3A–C). Likewise, immunofluorescence showed that P-Rex1 knockdown diminished TGF-β1–induced phosphorylation of TGFβR1 (Figures 3D,E). In contrast, a persistent and significant increase of TGFβR2 phosphorylation was observed after TGF-β1 stimulation in P-Rex1–expressing lung fibroblasts. Overexpression of P-Rex1 also slightly increased the phosphorylation of Smad2/3 compared to the vector control (Figures 3F–H). However, we found that overexpression of P-Rex1 increased the phosphorylation of TGFβR2 without TGF-β1 stimulation. P-Rex1 often acts upstream in the signal transduction pathway of cells. It seems that P-Rex1 may interact with TGFβR2 to participate in the TGF-β1 signaling pathway. P-Rex1 has been known for its multiple activation mechanisms. It is unclear how P-Rex1 expression regulated the activation of TGFβR2, but overexpression perturbed the cellular basal state, possessing its own disadvantages. Still, using gain-of-function experiments could play a supporting role in elucidating the role of P-Rex1 in TGF-β–mediated lung fibroblast migration and pulmonary fibrosis. In general, these data demonstrate that P-Rex1 expression is associated with the TGF-β1 signaling pathway.

### Association of P-Rex1 With TGFβR2 in Lung Fibroblasts

**FIGURE 4:**
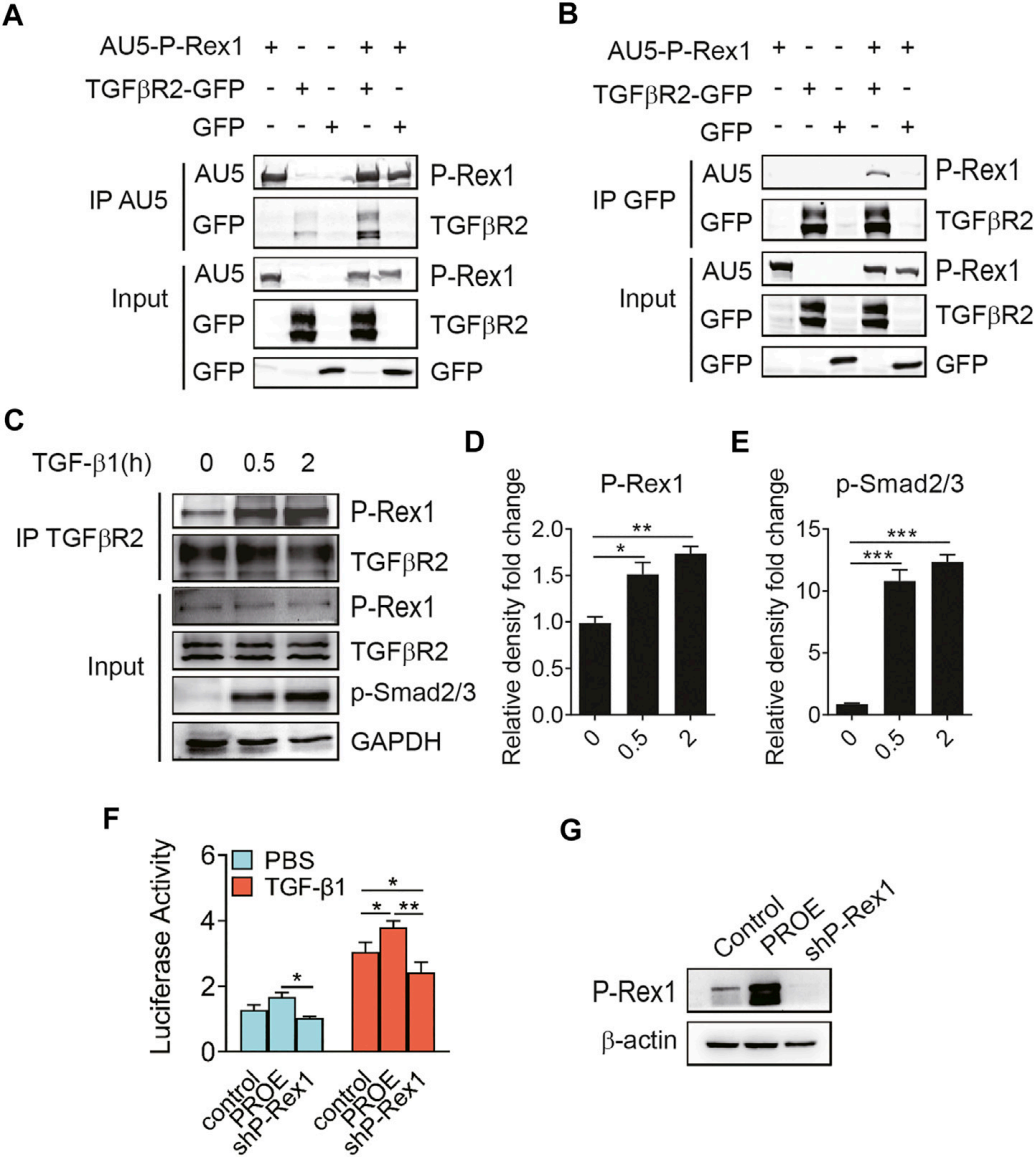
Association of P-Rex1 with TGFβR2 in lung fibroblast. **(A**, **B)** 293 T cells were transfected with AU5-tagged human P-Rex1, GFP-tagged TGFβR2, and GFP. Cell lysates were collected and subjected to immunoprecipitation using anti-AU5 antibody or anti-GFP antibody. Beads and total cell lysates were resuspended in loading buffer for analysis by Western blot. **(C)** Mouse lung fibroblasts were subjected to immunoprecipitation using an anti-TGFβR2 antibody post TGF-β1 (10 ng/ml) challenge. P-Rex1 expression was determined. **(D**, **E)** Quantification of data in **C**. **(F)** Luciferase activity of fibroblasts with P-Rex1 or shP-Rex1 transfection using SBE4-Luc reporter gene. **(G)** Expression of the P-Rex1 in fibroblasts. The data are shown above as mean ± SEM from three independent experiments. (*p < 0.05, **p < 0.01, and ***p < 0.001).

To further explore the P-Rex1 effector mechanism in TGF-β1 signaling, 293T cells were co-transfected with AU5-tagged P-Rex1 and GFP-tagged receptors for TGF-β1 signaling to establish a heterologous expression system, followed by a biphasic immunoprecipitation. As shown in Figures 4A,B, P-Rex1 and TGFβR2 formed a protein complex, indicating that P-Rex1 could interact with TGFβR2. Furthermore, TGF-β1 led to a significant increase of binding endogenous P-Rex1 to TGFβR2 in lung fibroblasts, suggesting a physical association between TGFβR2 and P-Rex1 (Figures 4C–E). Additionally, we conducted dual luciferase reporter assay using a Smad binding element 4 plasmid (SBE4-Luc) to confirm the involvement of P-Rex1 in TGF-β1/Smad signaling. We identified a notably upregulated luciferase activity in P-Rex1–expressing lung fibroblasts compared with control cells after treatment of TGF-β1. P-Rex1 knockdown significantly attenuated luciferase activity both at basal condition and TGF-β1 responsiveness (Figures 4F,G). Taken together, these findings indicate that P-Rex1 may be involved in TGF-β1 signaling in lung fibroblasts through interacting with TGFβR2.

### Identification of the Role of P-Rex1 in Lung Fibroblasts Using Next-Generation Sequencing

**FIGURE 5:**
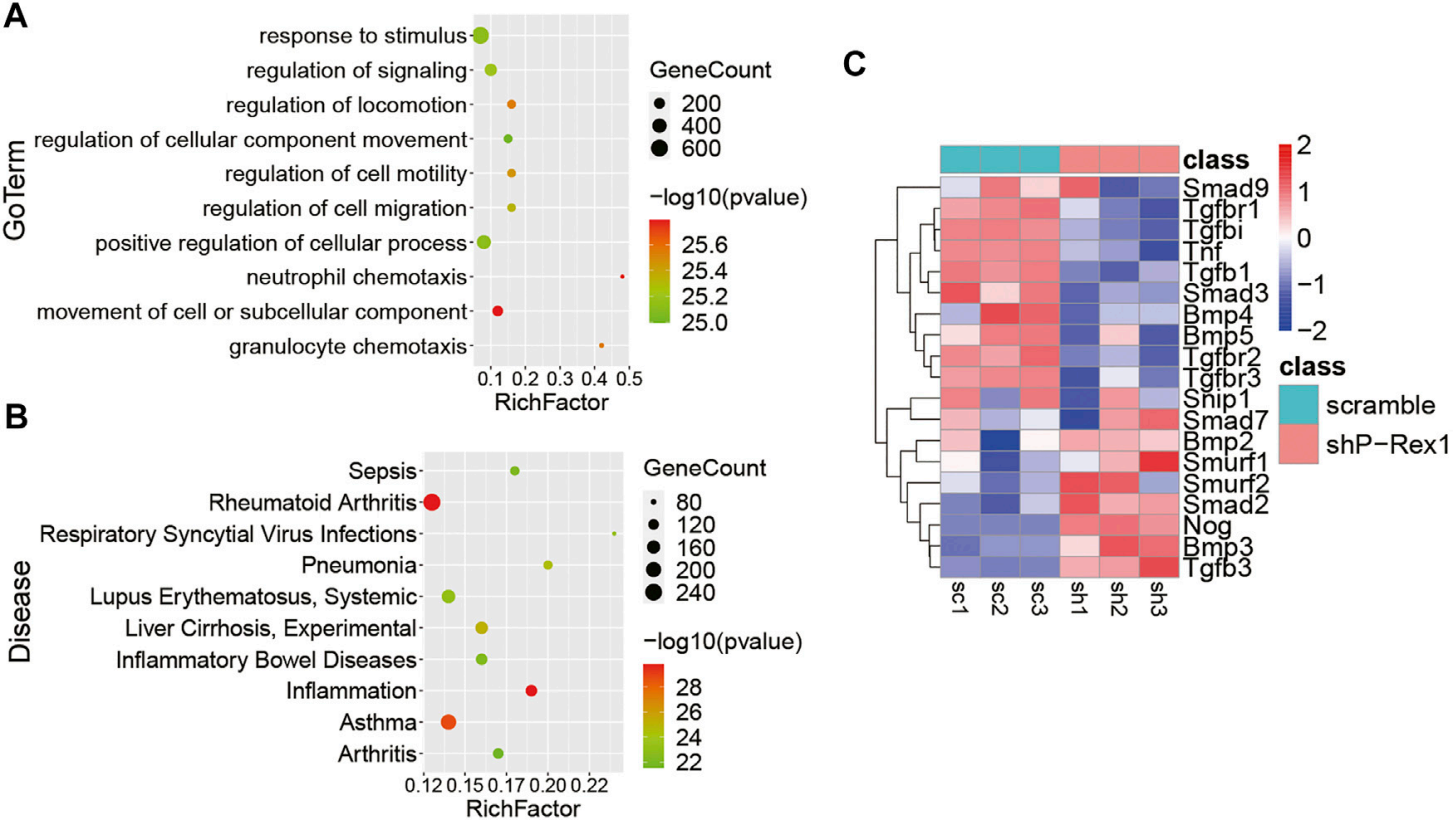
Identification of the role for P-Rex1 in lung fibroblasts using next-generation sequencing. **(A)** Gene Ontology (GO) enrichment showing top changes of cellular functions in P-Rex1 downregulated fibroblasts compared to control cells. **(B)** Bubble chart showed the top disease enrichment associated with P-Rex1 downregulation in lung fibroblasts. **(C)** Heatmap diagram showed the expression of Smad-dependent TGF-β1 signaling pathway related genes in P-Rex1 knockdown and scrambled control fibroblasts.

P-Rex1 knockdown mice fibroblasts and the scramble controls were applied the RNA-seq analysis for expression profiling to verify the cellular function and molecular mechanism of P-Rex1 in fibroblasts. Gene Ontology (GO) enrichment suggested that P-Rex1 mainly modulated cell motility and migration in molecular function, corresponding to our cellular function investigation (Figure 5A). Kyoto Encyclopedia of Genes and Genomes (KEGG) enrichment indicated that P-Rex1 appeared to be associated with inflammation or inflammation-related diseases (Figure 5B). These findings were consistent with the reported role for P-Rex1 in inflammation. Most Smad-dependent TGF-β1 signaling pathway related genes were identified as showing downregulated expression in P-Rex1 knockdown fibroblasts compared to the scramble controls. Specifically, there was viewed decreased expression of canonical Tgfb1, Smad3, Tgfbr1, Tgfbr2, and Tnf. Conversely, Smad7 and Smurf, acting as inhibitory TGF-β family signaling, were found significantly induced after P-Rex1 depleted (Figure 5C). Our data established a novel player of P-Rex1 in pulmonary fibrosis through involvement in TGF-β1 signaling that leading to changed fibroblast migration.

## Discussion

The P-Rex family is Dbl-type Rac-GEFs modulated by both Gβγ subunits of heterotrimeric G proteins and lipid second messenger phosphatidylinositol (3, 4, 5)-trisphosphate (PIP_3_). P-Rex1 is the first molecule of the P-Rex family originally identified from neutrophils (Welch et al., 2002). It contains 1,659 amino acids, and the isolated DH/PH domains tandem is the core of the GEF activity. P-Rex1 is involved in extensive biological procedures including ROS formation (Nie et al., 2010), platelet aggregation (Qian et al., 2012), chemotaxis (Marotti et al., 2017), endothelial permeability, pulmonary fibrosis, and cancer metastasis (Srijakotre et al., 2017). Our work unveiled a new role of P-Rex1 in the pathogenesis of pulmonary fibrosis. Increased expression of P-Rex1 was identified in lung tissues, peripheral whole blood, and myofibroblasts from pulmonary fibrosis compared to normal controls. P-Rex1 contributed to mouse lung fibroblast migration by acting as a downstream mediator of TGF-β1 signaling. TGF-β is mainly synthesized and secreted by alveolar macrophages and epithelial cells in the lung tissue. The TGF-β/Smad signaling pathway belongs to the transmembrane receptor with intrinsic serine/threonine kinase activity. TGF-β interacts with type I and type II receptor combination, resulting in the activation of intracellular effector Smad proteins and translocation into the nucleus, where they regulate gene transcription (Saito et al., 2018b). In pulmonary fibrosis, TGF-β1 regulates the recruitment of fibroblasts to the site of tissue injury and stimulates the differentiation of fibroblasts into myofibroblasts, which secrete extracellular matrix.

The effects of various cytokines are not separate that they interact and form a complex cytokine network. Other kinase pathways also modulate TGF-β/Smad signaling. Evidence has shown that Smad2 phosphorylation could result from the activation of epidermal growth factor (EGF) and hepatocyte growth factor (HGF) (Kim et al., 2016; Liu et al., 2019). Some mitogen-activated protein kinase (MAPK) pathways in response to receptor tyrosine kinases (RTKs) regulate Smad activation, while others might *via* Smad-independent signaling. Hyperactive oncogenic Ras/MAPK signaling led to impaired Smad signaling in tumor cells, suggesting a link between Ras and TGF-β response (Ichise et al., 2014). JNK, Erk, and p38 MAPK signaling could be activated by various stimuli. TGF-β1–induced activation of p38 MAPK induced the autocrine regulation of TGF-β1 that promoted epithelial–mesenchymal transition (EMT). Using Smad4-deficient cells, Engel et al. (1999) demonstrated that rapid activation of JNK in response to TGF-β1 implicated Smad-independent regulation and sustained activity suggested Smad-dependent. Crosstalk of various Rho GTPases with TGF-β signaling supported the possibility of targeting specific Rho GTPases as a potential strategy for TGF-β–dependent fibrosis. More recent studies have shown that Rac1 activation contributed to the formation of fibrosis (Patel et al., 2019). Blocking Rac1 signaling attenuated TGF-β1–induced fibroblasts migration (Ungefroren et al., 2018). Hereby, our results first indicated a correlation between P-Rex1, a GEF for Rho GTPase subfamily Rac, and TGF-β signaling. P-Rex1 contributed to the fibrogenic processes by acting as a downstream mediator of TGF-β1 signaling.

P-Rex1 is primarily identified as a downstream effector of GPCR signaling, but also acts as a mediator of ErbB signaling in cancer cell metastasis. P-Rex1 has been implicated in receptor tyrosine kinase pathways and sensitive to phosphatidylinositol 3-kinase (PI3K) inhibitor. (Ebi et al., 2013; Dillon et al., 2015; Marotti et al., 2017). PI3K was required for TNF-α–induced P-Rex1/Rac activation in the lung vascular permeability, suggesting a non-GPCR activation pathway for P-Rex1 (Naikawadi et al., 2012). A recent study has shown that P-Rex1 was involved in VEGF/VEGFR-dependent resistance in prostate tumors (Goel et al., 2016). These findings place P-Rex1 downstream of the RTK signaling pathway as a component of the positive feedback loop to drive RTK and PI3K. TGF-**β**1/Smad signal is often mediated through heteromeric type I and type II receptors. The involvement of GEF in the TGF-β/Smad signaling pathway is rarely reported. As we know, RIN1, a RAB5 guanine nucleotide exchange factor, could facilitate TGF-β receptor internalization and enhance TGF-β signaling (Hu et al., 2008). P-Rex1 appeared to cooperate with the platelet-derived growth factor receptor (PDGFRβ) to drive fibroblast migration (Campbell et al., 2013). This finding has expanded the research field of ideas on the mechanism of P-Rex1. Using the heterologous and endogenous co-immunoprecipitation model, we identified a direct or indirect interaction of P-Rex1 with TGFβR2 in lung fibroblast. TGF-β1 promoted the recruitment of P-Rex1 to TGFβR2 independently of GPCRs. Dual luciferase reporter assay confirmed the involvement of P-Rex1 in TGF-β1/Smad signaling. Of note, P-Rex1 is primarily involved in the regulation of inflammatory response. P-Rex1 deficiency attenuated bleomycin-induced lung inflammatory response. A combination of TGF-β1 signal pathway inhibitors with P-Rex1 inhibitor would shed light on therapeutic approaches for better pulmonary fibrosis treatment (Lucato et al., 2015; Cash et al., 2020).

Pulmonary fibrosis is an unmet public health need that demands continued research efforts. The identification of P-Rex1 as a component involved in the TGF-β1 signal pathway may contribute to elucidate the pathological mechanism of pulmonary fibrosis. Moreover, inhibition of P-Rex1 may be a potential strategy to target pulmonary fibrosis, but further investigation is required.

## Data Availability

The datasets presented in this study can be found in online repositories. The name(s) of the repository/repositories and accession number(s) can be found below: https://www.ncbi.nlm.nih.gov/, PRJNA718018
